# Addition of Platelet-Rich Plasma to Endorectal Advancement Flap Repair Does Not Enhance the Healing of Cryptoglandular Transsphincteric Fistulas

**DOI:** 10.1097/DCR.0000000000003312

**Published:** 2024-05-03

**Authors:** Michiel T.J. Bak, Jeanine H.C. Arkenbosch, Marte A.J. Becker, C. Janneke van der Woude, Annemarie C. de Vries, W. Rudolph Schouten, Oddeke van Ruler

**Affiliations:** 1 Department of Gastroenterology and Hepatology, Erasmus University Medical Center, Rotterdam, the Netherlands; 2 Department of Gastroenterology and Hepatology, Amsterdam Gastroenterology and Metabolism, Tytgat Institute for Liver and Intestinal Research, Amsterdam University Medical Center, University of Amsterdam, Amsterdam, the Netherlands; 3 Department of Surgery, IJsselland Hospital, Capelle aan den IJssel, the Netherlands; 4 Department of Surgery, Erasmus University Medical Center, Rotterdam, the Netherlands

**Keywords:** Cryptoglandular fistula, Perianal fistula, Platelet-rich plasma, Surgery

## Abstract

**BACKGROUND:**

Endorectal advancement flap repair is often performed for the treatment of cryptoglandular transsphincteric fistulas. However, this procedure fails in approximately 1 of 4 patients. Based on its supposed healing properties, platelet-rich plasma might enhance the outcome of this procedure.

**OBJECTIVE:**

To evaluate and compare the short-term and long-term outcomes after endorectal advancement flap repair with and without platelet-rich plasma injection in patients with a cryptoglandular transsphincteric fistula.

**DESIGN:**

Retrospective cohort study.

**SETTING:**

Tertiary referral hospital for proctology in the Netherlands.

**PATIENTS:**

Consecutive patients with a cryptoglandular transsphincteric fistula. Inverse propensity score–weighted comparison was used to adjust for confounding and selection bias.

**INTERVENTIONS:**

Endorectal advancement flap repair with and without platelet-rich plasma injection.

**MAIN OUTCOME MEASURES:**

Clinical fistula closure within 1 year without need for a reintervention (primary healing), clinical fistula closure within 1 year corrected for reinterventions (secondary healing), overall fistula healing within 1 year, and long-term outcomes assessed by a questionnaire.

**RESULTS:**

In total, 219 patients underwent an endorectal advancement flap repair. In 88 patients (40.2%), platelet-rich plasma was injected. No significant difference was observed in primary healing (67.0% vs 69.5%, *p* = 0.71), secondary healing (37.5% vs 43.5%, *p* = 0.60), or overall healing (73.9% vs 77.1%, *p* = 0.58) between patients treated with and without platelet-rich plasma injection. Long-term follow-up was available in 67.1% of the patients with a mean follow-up of 6.8 years (SD: 3.7 years). Among all patients who reached fistula healing, whether primary or secondary, within 1 year and had available long-term follow-up data, recurrence rates were also not significantly different (6.3% vs 2.9%, *p* = 0.37). Propensity score–weighted analysis showed that patients treated with a platelet-rich plasma injection were not more likely to achieve primary healing (OR 1.0; 95% CI, 0.5–1.9), secondary healing (OR 1.1; 95% CI, 0.2–3.2), overall healing (OR 0.9; 95% CI, 0.5–1.7), or recurrence at long-term follow-up (OR 1.1; 95% CI, 0.4–18.8) compared with patients without platelet-rich plasma injection.

**LIMITATIONS:**

Retrospective design, lack of postoperative imaging, and assessment of long-term follow-up using a questionnaire.

**CONCLUSION:**

Addition of platelet-rich plasma injection does not improve the short-term and long-term outcomes of endorectal advancement flap repair in patients with a cryptoglandular transsphincteric fistula treated in a tertiary referral center. See **Video Abstract**.

**ADICIÓN DE PLASMA RICO EN PLAQUETAS A LA REPARACIÓN DEL COLGAJO DE AVANCE ENDORRECTAL NO MEJORA LA CURACIÓN DE LAS FÍSTULAS TRANSESFINTERIANAS CRIPTOGLANDULARES:**

**ANTECEDENTES:**

La reparación con colgajo de avance endorrectal a menudo se realiza para el tratamiento de fístulas transesfinterianas criptoglandulares. Sin embargo, este procedimiento falla en aproximadamente uno de cada cuatro pacientes. Basándose en sus supuestas propiedades curativas, el plasma rico en plaquetas (PRP) podría mejorar el resultado de este procedimiento.

**OBJETIVO:**

Evaluar y comparar los resultados a corto y largo plazo después de la reparación con colgajo de avance endorrectal con y sin inyección de PRP en pacientes con una fístula transesfintérica criptoglandular.

**DISEÑO:**

Estudio de cohorte retrospectivo.

**ÁMBITO:**

Hospital terciario de referencia para proctología en los Países Bajos.

**PACIENTES:**

Pacientes consecutivos con fístula transesfintérica criptoglandular. Se utilizó una comparación ponderada por puntuación de propensión inversa para ajustar los factores de confusión y el sesgo de selección.

**INTERVENCIONES:**

Reparación del colgajo de avance endorrectal con y sin inyección de PRP.

**PRINCIPALES MEDIDAS DE VALORACIÓN:**

Cierre clínico de la fístula dentro de un año sin necesidad de reintervención (cicatrización primaria), cierre clínico de la fístula dentro de un año corregido por reintervenciones (cicatrización secundaria), curación general de la fístula dentro de un año y resultados a largo plazo evaluados mediante un cuestionario.

**RESULTADOS:**

En total, 219 pacientes se sometieron a una reparación con colgajo de avance endorrectal. En 88 pacientes (40,2%) se inyectó PRP. No se observaron diferencias significativas en la curación primaria (67,0% frente a 69,5%, p = 0,71), curación secundaria (37,5% frente a 43,5%, p = 0,60) y curación general (73,9% frente a 77,1%, p = 0,58).) entre pacientes con y sin inyección de PRP, respectivamente. El seguimiento a largo plazo estuvo disponible en el 67,1% de los pacientes con un seguimiento medio de 6,8 años (desviación estándar: 3,7 años). Dentro de todos los pacientes que alcanzaron la curación de la fístula, tanto primaria como secundaria, dentro de un año y tenían datos de seguimiento a largo plazo disponibles, las tasas de recurrencia tampoco fueron significativamente diferentes (6,3% vs. 2,9%, p = 0,37). El análisis ponderado por puntuación de propensión mostró que los pacientes tratados con una inyección de PRP no tenían más probabilidades de lograr la curación primaria (odds ratio [OR] 1,0; intervalo de confianza [IC] del 95 %: 0,5 – 1,9), curación secundaria (OR 1,1; IC del 95 % 0,2 – 3,2), curación general (OR 0,9; IC 95 % 0,5 – 1,7) o recurrencia en el seguimiento a largo plazo (OR 1,1; IC 95 % 0,4 – 18,8) en comparación con pacientes sin inyección de PRP.

**LIMITACIONES:**

Diseño retrospectivo, falta de imágenes postoperatorias y evaluación del seguimiento a largo plazo mediante un cuestionario.

**CONCLUSIÓN:**

La adición de la inyección de PRP no mejora el resultado a corto y largo plazo de la reparación con colgajo de avance endorrectal en pacientes con una fístula transesfintérica criptoglandular tratados en un centro de referencia terciario. *(Traducción— Dr. Ingrid Melo*)

Transsphincteric fistulas of cryptoglandular origin, especially those crossing the upper two-thirds of the external anal sphincter, are difficult to treat and (often) require sphincter-preserving procedures to minimize the risk of fecal incontinence. Various sphincter-preserving procedures have emerged for the treatment of these fistulas, including endorectal advancement flap repair.^1,2^ Despite many attempts to improve the outcome after endorectal advancement flap repair, pooled data reported an overall weighted success rate of approximately 75%.^2^

The development and persistence of perianal fistulas are thought to involve a complex interaction between histological, microbiological, and molecular factors, as fistula tracts have shown an increased expression of proinflammatory cytokines.^3,4^ Tissue regeneration is mediated by different intracellular and extracellular processes regulated by signaling proteins.^5^ Platelets are thought to be involved in wound healing and tissue repair as they can secrete multiple growth factors and factors involved in angiogenesis. In vitro studies have shown a dose–response relationship between platelet concentration and proliferation of mesenchymal stem cells (MSCs), fibroblast, and type I collagen.^6^ Furthermore, treatment with autologous platelet concentrate enhanced and accelerated wound healing in various animal models.^7^

Based on the hypothesis that platelets may improve both wound healing and tissue regeneration, an injection of platelet-rich plasma (PRP) as an add-on to endorectal advancement flap repair was introduced in our institution to improve the outcomes of endorectal advancement flap repair. A recent published meta-analysis, with data from 14 studies including 514 patients, reported an overall healing rate of 83.1% after local injection of PRP with additional surgical procedures, including endorectal advancement flap repair, ligation of the intersphincteric fistula tract, negative pressure wound therapy, and porcine collagen paste.^8^ Only two of the included studies reported the outcomes of endorectal advancement flap repair in combination with a PRP injection. The clinical closure rate at a 1-year follow-up was 90%. Their follow-up study reported a healing rate of 83% at a 2-year follow-up.^9,10^ However, none of these studies compared the outcome after endorectal advancement flap repair without PRP injection. Therefore, these two studies did not answer the question of whether the addition of PRP enhances the outcome of flap repair.^9,10^

In the absence of a head-to-head randomized controlled trial, our study aimed to compare short-term and long-term healing rates after endorectal advancement flap repair with and without PRP injection in a propensity score–weighted cohort study that included patients with a cryptoglandular transsphincteric fistula.

## MATERIALS AND METHODS

### Study Design

In this retrospective cohort study conducted in a tertiary referral center, consecutive adult patients (aged 18 years or older) with a cryptoglandular transsphincteric fistula and a single internal fistula opening who underwent an endorectal advancement flap repair between November 2005 and August 2019 were included. Exclusion criteria consisted of anovaginal fistula, presence of Crohn’s disease at the time of surgery or diagnosis during follow-up, absence of a detected fistula tract at the time of surgery, and/or absence of clinical follow-up data.

All flap repairs were performed by 1 experienced colorectal surgeon (W.R.S.). Before the procedure, MRI was performed on all patients. At the end of 2013, PRP injection was introduced to improve the outcome of endorectal advancement flap repair.

### Clinical Data Collection

Data regarding preoperative and postoperative visits to the outpatient clinic as well as the type of fistula surgery were retrospectively collected. These data included patient-related characteristics (eg, age, sex), disease-related characteristics (eg, type of fistula tracts, number of external openings, presence of multiple tracts), and surgical characteristics (eg, seton placement during surgery, postoperative complications within 30 days).

### Outcomes

The primary end point was clinical fistula closure, defined as complete closure of the external opening(s) without any fluid discharge from the fistulous tract at clinical examination, within 1 year following endorectal advancement flap repair without the need for a reintervention (ie, primary fistula healing). Secondary end points were clinical fistula closure within 1 year corrected for reinterventions (ie, secondary healing), overall fistula healing within 1 year, and long-term recurrence after initial fistula healing. Reinterventions included seton placement, fistulotomy, incision and drainage of perianal abscess, or repeat endorectal advancement flap repair with or without PRP injection. To evaluate the long-term outcomes, all patients received a standardized questionnaire between September 2019 and June 2020. For patients who had reached fistula clinical closure within 1 year, long-term fistula recurrence was defined as the need for a reintervention in the period thereafter.

### Surgical Procedure

Endorectal advancement flap repair was performed in patients with a high transsphincteric fistula, crossing the middle or upper one-third of the external anal sphincter. Flap repair was also performed in case of a failure after prior endorectal advancement flap repair or ligation of the intersphincteric fistula tract (LIFT) and/or in female patients with a low transsphincteric fistula of which the external opening was located anteriorly. During endorectal advancement flap repair, a Lone Star retractor (Lone Star Medical Products, Inc., Houston, TX) was used to expose the internal opening with the purpose of curating the internal opening. The remaining portions of the anal gland were excised. The internal opening was then sutured with a 3.0 or 4.0 Vicryl suture. The base of the flap comprised approximately one-third of the circumference, consisting of the mucosa, submucosa, and some of the most superficial fibers of the internal anal sphincter, to guarantee blood supply of the apex of the flap. The base measured approximately twice the width of the apex. Subsequently, the apex of the flap was raised from the level of the dentate line and mobilized over a distance of 4 to 6 cm proximally. The flap was advanced and sutured over the closed internal opening to the neo-dentate line with absorbable sutures 2/0 Monocryl (Ethicon Inc, Somerville, NJ). The external opening was cored out to the level of the external anal sphincter and left open to provide adequate drainage. On the discretion of the treating surgeon, preoperative drainage was performed with a seton or drain placement before the flap procedure in patients with associated abscess(es) and/or perianal sepsis detected on preoperative MRI.

To obtain autologous PRP, 15 mL of whole blood was drawn in a double syringe (Arthrex©) from the patient during endorectal advancement flap repair and centrifuged (1500 rpm for 4 minutes), after which 4 to 5 mL PRP was obtained from the upper (plasma) layer. PRP was injected around the internal opening and through the debrided external opening(s) into all quadrants in the tissue along the wall of the fistula tract.^11,12^

### Statistical Analysis

Descriptive statistical analyses (frequency, percentage, mean, SD, median, and interquartile range) were used to describe the research sample. Categorical variables were quoted as the number and percentage. Continuous variables were tested for normality using the Shapiro-Wilk test. Normally distributed variables were presented as mean (SD), whereas nonnormally distributed variables were presented as median (interquartile range). Differences between the two groups (PRP versus no PRP) were assessed using the χ^2^ test for categorical data and the independent *t* test for normally distributed continuous data.

Propensity score–weighted analysis was performed to adjust for baseline patient characteristics between the two groups. The inverse probability of treatment weighting (IPTW) method was chosen to retain all included patients in estimating the treatment effects and preserving statistical power.^13^ Propensity scores were calculated with the use of a multiple logistic regression model, in which treatment assignment PRP injection or no PRP injection was regressed on the basis of the following covariates/potential confounding factors: age at surgery, sex, complexity of the fistula (determined by the presence of side branches as seen on the preoperative MRI), prior attempts for repair (prior endorectal advancement flap repair and/or LIFT), and the need to perform an additional drainage during the procedure. Weighting was performed using IPTW. Analysis using IPTW are referred to as weighted analyses, while analyses in the unweighted cohort are referred to as unadjusted analyses. The statistical analysis of data was performed using IBM SPSS Statistics for Windows (version 25.0; IBM Corp, Armonk, NY).

### Ethical Consideration

This study was performed in accordance with the 2008 Declaration of Helsinki. This study was reviewed and approved by the local Medical Ethical Committee (MEC-2019-0522). Written informed consent was collected before inclusion in this study.

## RESULTS

Between November 2005 and August 2019, a total of 219 patients with a cryptoglandular transsphincteric fistula underwent endorectal advancement flap repair and were included in the present study. There were 40.2% of patients (n = 88) who received a PRP injection. Baseline characteristics are presented in Table 1. The majority of the patients were men (70.3%) with a mean age at surgery of 46.3 (SD: 11.9) years. A high transsphincteric fistula was present in 89.0% of the patients. Multiple fistula side tracts were present in 61.7% of patients. Twenty-six percent of the patients had undergone a previous attempt at fistula repair.

**TABLE 1.** Baseline characteristics in patients with cryptoglandular transsphincteric fistula treated with endorectal advancement flap repair with and without PRP injection

*Characteristics*	*Entire cohort (N = 219*)	*PRP (N = 88*)	*Non-PRP (N = 131*)	*p*
Male sex, n (%)	154 (70.3)	67 (76.1)	87 (66.4)	0.12
Age, y, mean (SD)	46.3 (11.9)	45.4 (12.7)	46.8 (11.3)	0.39
Female patients with an anterior internal fistula opening, n (%)	23 (35.4)	10 (47.6)	13 (29.5)	0.15
High transsphincteric fistula,^a^ n (%)	195 (89.0)	72 (81.8)	123 (93.9)	0.01
Presence of multiple fistula side tracts, n (%)	129 (61.7)	48 (57.1)	81 (64.8)	0.26
Preoperative seton, n (%)	46 (21.0)	32 (38.1)	14 (11.2)	<0.01
No. of external fistula openings, n (%)				0.64
1	182 (86.7)	73 (84.9)	109 (87.9)	–
2	27 (12.9)	12 (14.0)	15 (12.1)	–
3	1 (0.5)	1 (1.2)	–	–
Previous fistula surgery, n (%)	204 (93.2)	84 (95.5)	120 (91.6)	0.27
Previous attempt for fistula closure, n (%)	57 (26.0)	31 (35.2)	26 (19.8)	<0.01
Endorectal advancement flap repair	44 (20.1)	25 (28.4)	19 (14.5)	–
LIFT	17 (7.8)	10 (11.4)	7 (5.3)	–

In case of missing data, valid percentages are presented.

LIFT = ligation of the intersphincteric tract; PRP = platelet-rich plasma.

a A high transsphincteric fistula was defined as a fistula crossing the upper two-thirds of the external anal sphincter on MRI.

### Surgical Characteristics

During endorectal advancement flap repair, a temporary drain was placed in 13.3% of the patients (23.9% treated with PRP vs 5.7% treated without PRP) by contraincision to improve drainage in case of extensive side branches or cavitation. A Malecot catheter was used for rinsing in 39.7% of patients (23.5% with PRP vs 52.3% without PRP) to improve drainage in case of extensive side branches or cavitation. No adverse events, regarding PRP injection, were reported. Postoperative complications after endorectal advancement flap repair comprised bleeding (4.2%; n = 9), all treated with a conservative approach; postoperative abscess (3.3%; n = 7), all treated with incision and drainage; and flap dehiscence (0.5%; n = 1), needing an additional flap repair.

### Clinical Outcomes After Endorectal Advancement Flap Repair With or Without PRP Injection

Overall primary healing, at 1 year of endorectal advancement flap repair, was observed in 68.5% of patients (n = 150; Table 2). Comparing patients with and without PRP injection, the fistula healing rate was found to be similar (67.0% vs 69.5%, *p* = 0.71) in the unweighted analysis. A reintervention was performed within 1 year after endorectal advancement flap repair in 39 patients (17.8%; 16 patients treated with PRP [18.2%] vs 23 patients treated without PRP [17.6%], *p* = 0.94) because of healing failure. Of these 39 patients, secondary healing was observed in 16 patients (43.6%; 6/16 patients treated with PRP [37.5%] vs 10/23 patients without PRP [43.5%], *p* = 0.60), resulting in a 1-year overall healing rate in 75.8% of patients (n = 166). The remaining patients (n = 31), without healing within 1 year after surgery, did not undergo reintervention within 1 year for various reasons.

**TABLE 2.** Outcomes on clinical closure and reinterventions

*Outcomes*	*Entire cohort (N = 219*)	*PRP (N = 88*)	*Non-PRP (N = 131*)
Primary healing, n (%)	150 (68.5)	59 (67.0)	91 (69.5)
Secondary healing, n (%)	17 (38.5)	6 (31.3)	10 (43.5)
Overall healing, n (%)	166 (75.8)	65 (73.9)	101 (77.1)
Total number of unplanned reinterventions,^a^ n	18	7	11
Endorectal advancement flap repair with PRP	4	3	1
Endorectal advancement flap repair	9	1	8
Seton placement	2	1	1
Fistulotomy with PRP	1	1	0
Fistulotomy	1	1	0
Incision and drainage	1	0	1
Long-term follow-up available, n (%)	147 (67.1)	65 (73.9)	85 (64.9)
Mean time of follow-up, y, n (%)	6.8 (3.7)	3.7 (1.3)	9.2 (3.1)
Long-term recurrence,^b^ n (%)	5 (3.4)	3 (6.3)	2 (2.9)

PRP = platelet-rich plasma; PRS = platelet-rich stroma.

a Unplanned reinterventions (<1 y) are only presented for patients who achieved clinical closure.

b Long-term recurrence was assessed for patients who reached overall healing within 1 y.

No significant difference in overall healing rate at 1 year after endorectal advancement flap repair was observed between patients treated with PRP (73.9%) and patients treated without PRP (77.1%; *p* = 0.58). Propensity score–weighted analysis showed that patients treated with PRP injection were not more likely to achieve primary healing (OR 1.0; 95% CI, 0.5–1.9), secondary healing (OR 1.1; 95% CI, 0.2–3.2), and consequently, also not for overall healing (OR 0.9; 95% CI, 0.5–1.7) at a 1-year follow-up.

### Long-term Follow-up After Endorectal Advancement Flap Repair

A total of 147 patients (67.1%) completed the long-term follow-up questionnaire (73.9% with PRP vs 64.9% without PRP, *p* = 0.16). The mean duration of follow-up was 6.8 years (SD: 3.7). Within the group that reached overall fistula healing within 1 year and with available long-term follow-up data, the overall recurrence rate was 3.4% and not significantly different between those treated with PRP (6.3%) and without PRP (2.9%, *p* = 0.37). Propensity score–weighted analysis showed that patients treated with PRP injection were not more likely to experience recurrence (OR 1.1; 95% CI, 0.4–18.8) after achieving fistula healing within 1 year.

## DISCUSSION

This large cohort study aimed to evaluate and compare the potential role of PRP injection in patients who underwent flap repair for a cryptoglandular transsphincteric fistula. Propensity scores were weighted in the analysis to adjust for potential confounding and selection bias. In both unweighted and weighted analyses, no beneficial effect of PRP injection was observed on both short-term and long-term outcomes of flap repair in patients with a cryptoglandular transsphincteric fistula.

The effects of PRP injection have been described in several medical disciplines, such as dentistry, cosmetic and orthopedic surgery, and dermatology.^14,15^ The effectiveness of PRP in the treatment of both cryptoglandular fistulas and fistulas because of Crohn’s disease has been studied in several small (pilot) studies as well as small randomized controlled trials. A recent meta-analysis reported an overall healing rate of 62.4% after PRP injection (with or without additional closure of the internal orifice with resorbable sutures) and 83.1% when combined with other approaches, including surgical procedures such as LIFT of endorectal advancement flap repair, but also porcine collagen paste and negative wound pressure therapy. However, these results should be interpreted with caution because of heterogeneity in fistula characteristics (cryptoglandular fistulas vs Crohn’s disease–related fistulas, additional fistula surgery vs no additional fistula surgery, and different types of fistula), the unclear definitions of the assessed outcomes, overall small sample sizes, and a short duration of follow-up.^8^ Only two of the included studies assessed the effectiveness of a PRP injection endorectal advancement flap repair in patients with a high cryptoglandular fistula.^9,10^ van der Hagen et al^9^ reported a clinical closure rate of 90% at 12 months after endorectal advancement flap repair with PRP injection in 10 patients with a high cryptoglandular fistula. In a follow-up study, including 25 patients, a healing rate of 83% at two years after endorectal advancement flap repair and PRP injection was observed.^10^ These results could not be reproduced by our present study, for which an unequivocal explanation is lacking. It may be possible that the patients, referred to our institution, presented with a more complex fistula, reflected by the higher number of fistulas with side branches (62% vs 10%) compared with the study of van Hagen et al.^9^ Our study also differs from the two studies, mentioned above, by the significantly larger cohort of patients and the much longer duration of follow-up. Although these studies reported an impressive result after flap repair with PRP, in both short-term and long-term, the authors did not compare the healing rates with patients who underwent flap repair without PRP.

Platelets are an important mediator in the process of wound healing by secreting growth factors and chemokines. These growth factors and chemokines facilitate the recruitment of inflammatory cells as monocytes and macrophages, promoting migration, proliferation, and activation of fibroblasts.^16^ As PRP contains a high number of growth factors, it is hypothesized that PRP can augment wound healing. Growth factors present in PRP include epidermal growth factors and transforming growth factors, and also angiogenic factors like vascular endothelial growth factor, platelet-derived growth factor, and fibroblast growth factors, which are secreted after thrombocyte activation.^17^ It is thought that PRP can only be effective when vital cellular components for regeneration are present (eg, vital cells around the fistula tract wall).^17^ Concerning perianal fistulas, the effect of PRP can be hampered by the complexity and extension of the fistula (defined by the presence of side branches and the need for additional drainage during surgery). However, no differences in propensity score–weighted analysis for both short-term and long-term outcomes were observed between patients with and without PRP injection after adjustment for the complexity and extension of the fistula using IPTW.

Recent studies have shown that PRP, in combination with autologous adipose-derived stromal cells (ie, stromal vascular fraction [SVF]), is feasible and effective for both cryptoglandular fistulas and Crohn’s disease–related fistulas.^11,12,18–20^ SVF is thought to augment wound healing through the additional presence of leukocytes, macrophages, and vascular endothelial cells, all embedded in fibrovascular network. It is thought that PRP can improve the healing properties of SVF.^21^ Although these results are promising, randomized studies are currently absent. Future (randomized) studies, including long-term follow-up, are warranted to explore the effectiveness of PRP with SVF. Furthermore, the use of MSCs as a treatment for cryptoglandular fistulas has been studied, in recent years, with promising results. A recent meta-analysis was conducted assessing clinical trials, which studied the efficacy MSCs in both cryptoglandular and Crohn’s disease–related perianal fistula.^22^ These pooled data reported a superior outcome in patients treated with MSCs treatment compared with the control group (fibrin glue, saline, or surgery) for closure of the external openings and/or absence of the drainage through these openings on short-term follow-up (≥3 months; RR 2.5; 95% CI, 1.6–33.8) and long-term follow-up (at ±1 year; RR 1.4; 95% CI, 1.1–1.7) and extended follow-up (>1 year; RR = 1.9; 95% CI, 1.1–3.1). However, in the studies with patients with cryptoglandular fistula, no beneficial effect could be observed between patients treated with MSCs and those in the control group (RR = 1.16; 95% CI, 0.7–2.0).^22^ Several randomized control trials assessing the efficacy of MSCs for the treatment of complex cryptoglandular fistulas are awaited (NCT05709717, NCT04750499).

To our knowledge, this study is the first to assess the effectiveness of PRP in addition to endorectal advancement flap repair in a large cohort study. However, this study has several limitations. First, this study is limited by its retrospective design. To address this limitation, we have used IPTW to adjust for potential confounding and selection bias.^13^ Second, no postoperative MR images were available in this study, limiting the information on complete healing of the fistula throughout the entire fistula tract. To date, the added value of postoperative MRI is unknown for cryptoglandular fistula. In recent literature regarding fistulizing Crohn’s disease, it is considered that clinical fistula closure does not always lead to complete radiographic fistula closure on MRI.^23^ Furthermore, no standardized data on potential confounding factors for failure of surgery, such as smoking, obesity, diabetes, and/or radiation, were present. Finally, the long-term follow-up was assessed using a questionnaire.

## CONCLUSION

Injection of PRP as an add-on to endorectal advancement flap repair does not improve the short-term and long-term outcome of endorectal advancement flap repair in patients with a cryptoglandular transsphincteric fistula treated in a tertiary referral center.

## ACKNOWLEDGMENTS

The authors thank the medical secretary of the Surgical Department of the IJsselland Hospital and Charlene van der Zijden, research intern, for assisting in the data collection.
